# TURP syndrome: à propos d’un cas

**DOI:** 10.11604/pamj.2017.28.243.9210

**Published:** 2017-11-17

**Authors:** Said Benlamkaddem, Nawfal Houari, Brahim Boukatta, Hicham Sbai, Nabil Kanjaa

**Affiliations:** 1 Service de Réanimation Polyvalente A4, Centre Hospitalier Universitaire Hassan II, Fès, Maroc

**Keywords:** Résection transuréterale de la prostate, TURP syndrome, rachianesthésie, hyponatrémie, Transurethral resection of the prostate, TURP syndrome, spinal anesthesia, hyponatremia

## Abstract

Nous rapportons le cas d'un patient de 78 ans, sans antécédents pathologiques notables, qui a bénéficié d'une résection transuréterale d'une hypertrophie bénigne de prostate de 50g sous rachianesthésie. Ce patient a présenté, 90 minutes après le début de l'intervention, des nausées vomissements, brouillard visuel et bradycardie en rapport avec un TURP syndrome. L'ionogramme a objectivé une natrémie à 118meq/l, d'où sa mise sous sérum salé hypertonique à 3% avec bonne évolution. Cette observation décrit une forme typique mais modérée du TURP syndrome dont la prise en charge était facilitée par l'état d'éveil du patient permis grâce à la rachianesthésie.

## Abstract (en)

We report the case of a 78-year old patient, with no particular past medical history, who underwent transurethral resection of the prostate (50 g) under spinal anesthesia for benign hypertrophy. 90 minutes after the beginning of the procedure, the patient had nausea, vomiting, visual fog and bradycardia, suggesting TURP syndrome. Ionogramme objectified a serum sodium level of 118meq/L, hence the patient was treated with 3% hypertonic saline solution, with good evolution. This study describes a common but moderate occurrence of TURP syndrome whose management was facilitated by patient’s alertness during spinal anesthesia.

## Introduction

La résection transuréterale de la prostate est une intervention courante chez l'homme après l'âge de 60 ans. Cette intervention, quoique simple, peut se transformer en une catastrophe à cause du TURP (Transurethralresection of the prostate) syndrome qui est dû à une absorption massive de liquide d'irrigation [1]. Ce syndrome peut entrainer une atteinte neurologique, un œdème aigue de poumon, voire des manifestations cardiovasculaires graves [2]. Son incidence varie entre 0,78% et 1,4% [3]. Sa prise en charge impose l'arrêt de l'intervention, la correction des troubles hémodynamique et respiratoire et la correction de l'hyponatrémie aigue avec du sérum salé hypertonique.

## Patient et observation

Nous rapportons le cas d'un patient de 73 ans sans antécédents pathologique notables, programmé pour une résection transuréterale de la prostate (RTUP ou TURP en Englais) pour une hypertrophie bénigne de la prostate d'un poids de 50g. L'examen clinique préopératoire, l'ECG et la radiographie thoracique étaient sans particularité ; la natrémie était à 137 meq/l, la kaliémie à 3,7meq/l, la fonction rénale était normale, l'hémoglobine était à 12,3g/dl, et l'examen cytobactériologique des urines (ECBU) était négatif. Le geste chirurgical était fait sous rachianesthésie avec 2,5 ml de bupivacaine 0,5% permettant l'obtention d'un bloc au niveau T10. Le patient était mis, ensuite, en position gynécologique pour la résection. Le liquide utilisé pour l'irrigation était la glycine 1,5%, la poche étant fixée à un niveau de 1m au-dessus de la table opératoire. Le volume total de liquide utilisé au cours du geste était de 12 litres sur une durée totale d'intervention de 120 minutes. La TURP était réalisée par un résectoscope monopolaire. Après 90 min du début de la résection, le patient avait présenté une agitation, des nausées, des vomissements, des vertiges, un brouillard visuel une bradycardie et une hypertension artérielle à 180/100 mmhg tout en gardant un état de conscience normal (un score de Glasgow à 15). L'auscultation pulmonaire n'avait pas objectivé de râles crépitant. L'ECG avait mis en évidence une bradycardie sinusale sans autres modifications électriques. Un prélèvement sanguin était fait et le patient était mis immédiatement sous 100 ml de sérum salé hypertonique à 3% et 20 mg de furosémide devant la suspicion de TURP syndrome. Le bilan biologique avait objectivée, par la suite, une natrémie à 118 meq/l, une kaliémie à 3,4 meq/l, une hémoglobine à 9,7g/dl et une troponine négative. Un deuxième ionogramme était fait après transfert du malade au service de la réanimation et 30 min après le premier bolus de SSH 3% et avait objectivé une natrémie à 120meq/l d'où un deuxième bolus de 100 ml de SSH 3%. La natrémie de contrôle était à 126 meq/l. le patient était mis, ensuite, sous sérum salé isotonique 2,5 litres/j et du furosémide 20 mg toutes les 8 heures. L'évolution était marquée par la disparition des symptômes avec, au bilan biologique de contrôle, une natrémie à 133 meq/l, une kaliémie à 3,6 meq/l, une fonction rénale normale et une hémoglobine à 8,8g/dl. Le patient était, ensuite, transféré au service d'urologie après 24 heures d'hospitalisation en réanimation.

## Discussion

Le TURP syndrome est une complication très connue de la résection transuréterale de la prostate. Elle résulte d'une absorption massive d'eau libre à partir du liquide d'irrigation responsable d'une hypervolémie et, surtout, d'une hyponatrémie [4]. Plusieurs facteurs favorisent la survenue de cette complication comme l'utilisation de soluté hypotonique pour l'irrigation (sorbitol, mannitol, eau distillée, glycine…), surtout en grande quantité, un volume de prostate dépassant les 60g, une durée d'intervention au-delà de 60min [5] et un niveau de la poche du liquide d'irrigation au-dessus de 60cm [6]. Le tableau clinique varie de la forme asymptomatique aux formes caractérisées par des nausées, vomissement, sensations de mouches volantes, de brouillard visuel, hypertension artérielle, crise convulsive, coma voire le décès [7]. Aucune étude n'a montrée de différence en matière de pertes sanguines, d'altération des fonctions supérieures postopératoires et de mortalité entre l'anesthésie générale et la rachianesthésie [8]. Cependant, la rachianesthésie permettrait de surveiller les signes neurologiques chez un patient réveillé, et ainsi, d'accélérer la prise en charge diagnostique et thérapeutique, comme c'est le cas chez notre patient qui a bénéficié d'une prise en charge rapide expliquant le caractère modéré des manifestations cliniques. Le traitement de cette complication dépend de la gravité du tableau clinique. Il peut nécessiter le recours à une assistance des fonctions vitale (ventilation mécanique, drogues vasoactives, expansion volémique') associée au traitement de l'hyponatrémie avec du sérum salé hypertonique à 3% sous forme de bolus de 100 ml à répéter en fonction de la natrémie et pour ramener la natrémie à 130 meq/l [9]. Plusieurs techniques permettent actuellement de prévenir le TURP syndrome comme l'utilisation d'un résectoscope bipolaire avec sérum salé comme soluté d'irrigation [10], la vaporisation au lieu de la résection prostatique et en fin, le contrôle de la pression hydrostatique au niveau de la loge prostatique qui dépend du niveau de la poche du liquide d'irrigation.

## Conclusion

Le TURP syndrome est une complication rare mais grave. La prévention de ce syndrome nécessite une parfaite connaissance des facteurs de risques, une bonne entente entre le réanimateur-anesthésiste et le chirurgien, et l'utilisation de techniques permettant de réduire le risque de saignement et, ainsi, l'absorption de liquide d'irrigation.

## Conflits d’intérêts

Les auteurs ne déclarent aucun conflit d’'intérêts.
